# Auditory discrimination improvement in young adults by a digital auditory game-based training protocol

**DOI:** 10.1371/journal.pone.0313478

**Published:** 2025-10-23

**Authors:** Sergio M-Cam, Luz Maria Alonso-Valerdi, David I. Ibarra-Zarate

**Affiliations:** Monterrey Institute of Technology and Higher Education (ITESM), Engineering and Sciences School, Monterrey, NL, Mexico; Center for Healthy Start Initiative, NIGERIA

## Abstract

Auditory training is a technique that has demonstrated to be beneficial, improving auditory processing response as a supplement to hearing loss management. Recently, game-based training approaches have also demonstrated to increase the effect of traditional auditory training. Hence, the general aim of this work is to develop a game-based auditory discrimination training to enhance the auditory processing response and measure performance in comparison to traditional methods. The evaluation of the methods will be in three directions: (1) user experience, (2) perceptual and behavioral responses, and (3) neurophysiological performance. For this purpose, the project will be conducted as follows: (1) to develop and validate a game-based auditory discrimination training method in line with the usability level, and (2) to evaluate the auditory training in terms of method performance, and neurophysiological responses, before and after the procedure. The game-based auditory training will be focused on basic sound features discrimination tasks, which will be applied for 15 hours with 4 different modules. This will contribute to understand experience processes that yield to a better training performance.

## Introduction

According to the World Health Organization (WHO) [1], hearing loss is defined as the condition in which a person cannot hear as well as a person with normal hearing (thresholds are 20 dB HL or better on both ears). It is considered the most common and prevalent sensory deficit in the world [2,3] with estimates of 360 million people in 2022, and 466 million people in 2023. For 2030, it is expected to be the 7th most common disease [4]. Hearing loss severity is divided from moderate, severe moderate, severe or profound depending on hearing thresholds. According to the Rehabilitation National Institute in Mexico [5] in 2021, there existed about 2.3 million people with hearing loss in Mexico and it is estimated that 3 of each 1000 newborn have hearing loss [6]. Some of these numbers have not been updated since the recent pandemic situation (COVID-19). It is speculated that one of these virus sequels is hearing loss. Significant increase of people with hearing loss is suggested after COVID-19 pandemic [7].

There exist different solutions and treatments for people with hearing loss such as hearing aids, bone conduction headphones, surgeons, cochlear implants, and other interventions depending on the hearing loss type and severeness. Once hearing loss is correctly managed following best diagnosis and intervention practices [8,9], these solutions require adaptation processes and rehabilitation therapies to optimize their results. Sometimes, these alternatives may also be either uncomfortable or painful and their costs may be high for the low-income population. Non-invasive procedures and non-destructive means for hearing loss population are needed [3].

Auditory Training (AT) has shown to improve auditory processing conditions in hearing loss and normal hearing population through sessions of habilitation and rehabilitation [10–12]. However, neural plasticity changes may be not restored completely as that in normal hearing individuals [3]. AT has been mostly implemented through traditional methodologies and techniques, where experience-dependent performance is uncommonly considered, using not entertaining or engaging tools and schemes. Although, it has been demonstrated that higher success and achievement levels are related to higher levels of immersion [13] Indeed, some new digital tools have been already proposed to enhance user experience in AT. These proposals have designed for dichotic and speech.

The usage of computerized-based and game-based training tools can manage emerging population necessities and commodities favoring accessibility to healthcare technology. People can have complementary training at home, optimizing audiologists time and theirs own, and reducing hospitals and clinics saturation, satisfying solutions as person-centered tools for tele-audiology [14].

Although there are some types of hearing loss that are not reversible and cannot be healed or cured yet by a medical treatment, such as permanent hearing loss due to conductive or some sensorineural damage, there are some others such as some sensorineural, sudden and mild hearing loss that can be recovered to a certain level or can be treated with some other devices. All of them require adaptative processes to assimilate new auditory reality, such as AT. Taking care of hearing has never been so important as now. At present, prolonged exposure to environmental noise, overuse of earphones and new appearing diseases such as COVID-19, hearing has become a public health problem. Regular audiological check-ups, consciousness about high sound intensities exposure, avoidance of harmful substances, are some of the considerations for hearing healthcare.

Over the past 3 years, COVID-19 was spread over the world with more than 7 hundred million cases [15], developing a diversity of physiological reactions leaving a series of sequels, some of them still unknown. As was mentioned above, one speculated derivation from this virus is hearing loss, due to a strong infection, it can yield to sudden sensorineural hearing loss [16]. During this pandemic period, access to hospitals and clinic visits were limited. Even some of those people with audiological treatments that have been in therapy before 2020, had seldom follow-up sessions. Considering this, new audiological reality establishes two main challenges. One of them is to having better tools for auditory adaptative processes in rehabilitation, and second one is to develop tools that enhance engagement tasks and active involvement of users to obtain better training performance.

### State of the art

#### Computerized AT performance.

AT has been mostly applied since the beginning of early 2000s, in this brief review some of the most important and relevant studies and their results will be summarized to give a general idea of latest research and their highlights.

Two main assessments for AT performance have been conducted. The first one was to measure an improvement in auditory defined tasks after AT and an evaluation pre and post training to be compared through tasks’ results and behavioral tests. These tasks involve audiological assessments and specific training techniques in each study. The second assessment involves electrophysiological response before and after AT, mostly based on auditory evoked potentials (AEPs). Fewer cases evaluated the training performance by some other tests, including some of them the usability and validation of their methodology.

There has been studied the effects of AT in auditory response and perception through different means and different methodologies and it has been demonstrated the effectiveness to improve auditory skills such as general sensory, social and cognitive skills [17], of people with hearing impairment, intervention rehabilitation and auditory disorders. There is a broad literature on AT employing a variety of training skills for different populations [18,19]. One recent approach has been incorporated to evaluate interfaces usability and experience in the effects of AT [20]. The efficiency of this structure set, as mentioned by the authors, depended mainly on auditory experience. Auditory tasks and exercises, even the simplest ones, are influenced by high-level functions such as memory, motivation and decision-making [12].

Barton and Brewer [12] indicate the following principles as important to follow during AT:

- Frequent, challenging and motivating.
- Diverse tasks to maintain motivation.
- Gradual in difficulty over time.
- Follow-up acquired responses (>70%).
- Monitored based on psychophysical, electrophysiological and questionnaire-based data.

It has also been demonstrated that long-term changes in auditory cortical neuronal single unit or population activity that is involved in sound acoustic experience mostly result from the differential engagement of similar neural mechanisms [21]. In this way, motivation and attention during activity has been measured through electrophysiological signal acquisition analyzing engagement index level beta/alpha correlation for sustained attention performances [22], confirming a relation between task engagement and alpha/beta rhythms.

#### AT and game-based training.

With the purpose of offering a better experience and motivation, game-based training approaches and computer-based AT have been little explored or have not always followed scientific guidelines for unbiased research. Gamification has enhanced engagement and learning, and studies about game-based tools and game element effects have reported an increase in user retention and other favorable results [23]. Research done at the beginning of 2010s has focused on offering computerized-based and digital tools for hearing and aural rehabilitation improving user accessibility and fitting new technology possibilities. Olson [24] provides review of specific computer and mobile-based AT programs considering their usefulness in patient centered care and self-management.

There have been some methodologies and developments to apply AT with computer or game-based approaches. For this research purpose, the three following approaches are described due to the similarity in methodology or evaluation.

One recent study by Stewart [25] tested auditory cognition and perception with 80 participants with a specific videogame experience, as well as visual reaction task, but performance of auditory tasks did not present significant benefits, suggested by visual environment interaction rather than auditory. Whitton tried to demonstrate that perception and learning on a computerized audio game can be developed after an eight-week AT program for the hearing-impaired population [13]. Whitton reported that after this time speech-in-noise intelligibility with conditions that challenges listening has improved about 25% and that control ability and game strategy predicted individual training improvement [26].

Tuz developed a computer-based auditory AT [20] evaluating 40 adults with hearing impairment proposing five levels of usability subscales: (1) ease of use, (2) comprehensibility, (3) design, (4) satisfaction and (5) motivation. They validated their development through the Software Usability Measurement Inventory (SUMI) and Computer Usability Satisfaction Questionnaires for usability evaluation purposes, in five main usability subscales. Later, they applied the computer-based AT evaluating nine auditory modules. Although their development and research were mainly focused in this first stage to design and validate the program, it addressed an antecedent in usability validation for AT resources, suggesting that validation obtained by both specialists and participants may be an indicator of retaining motivation of individuals.

One of the latest research projects studied learning transfer through an AT game for speech perception. Gamified mixed training approach was applied to college-aged with no hearing difficulties. Software training performance was evaluated across task progress, auditory perceptual outcomes, and cognitive outcomes, investigating the effectiveness of gamified AT on speech in competition and reporting improvement in working memory updating task [19]. Still, it was concluded that more research is required to understand which game elements these effects are dependent on as it is also suggested that game design elements motivation should be analyzed in specific contexts [27].

#### AT & auditory evoked potentials.

Since different techniques of AT have been developed, there has been various research to demonstrate the effectiveness of AT in electrophysiological measurements through AEP extraction and analysis.

Binaural AT effectiveness was evaluated comparing skills in three moments, before training, immediately after, and 3 months after. Participants 19–30 years old received training 30 min twice a week during 12–20 sessions. Long latency auditory evoked potentials (LLAEP), frequency following response and auditory brainstem response (ABR) were obtained showing subtle latency reduction in ABR components 3 months after AT and amplitude increase in left ear P3 component [28].

Another recent research measured auditory-based targeted cognitive training performance through mismatch negativity (MMN) and P3a components as neuromarkers of early auditory information processing. Auditory-based targeted cognitive training was implemented through exercises of auditory frequency discrimination. Training was implemented for 1 hr. just once and no significant changes were found between baseline and final auditory-based targeted cognitive training [29].

#### Aim.

In the light of the evidence, the aim of this research is to determine whether differences in user experience between the two systems; a game-based auditory discrimination training program and a non-game-based auditory discrimination training program, lead to differences in auditory discrimination perceptual, behavioral and electrophysiological performance within a normal-hearing population To meet this aim, the research will be conducted as follows:

- To develop and validate a digital game-based auditory discrimination training tool.
- To develop a weekly program and to monitor key performance indicators (KPIs) in terms of time, accuracy and discrimination thresholds.
- To measure auditory processing improvement through perceptual, behavioral and electrophysiological tests comparing discrimination tasks’ performance before and after the use of auditory discrimination training system.

## Methods

### Sample size, inclusion & exclusion criteria

Sample Size is determined by two means; literature has worked with sample sizes averages between 10–40 participants, due to the long training period and evaluation requirements [30,31]. Sample size was calculated determining F-tests family for ANOVA repeated measures between and within interactions, given a moderate effect size of 0.25, alpha error prob of 0.05 and power of 0.80, two groups and two measurements. Effect size was determined with related previous works, where effect sizes of behavioral auditory discrimination measurements are reported from moderate to large, based on Cohen’s d and Cohen’s f calculations [32–35]. Larger effects sizes have been suggested when individuals were submitted for first time to auditory devices [36].

G*Power 3.1 points a total sample size for both groups of 34. Considering two independent groups for auditory discrimination training (ADT) implementation, sample size for Group A (control), and Group B (experimental) will be equally distributed.

**Fig 1:**
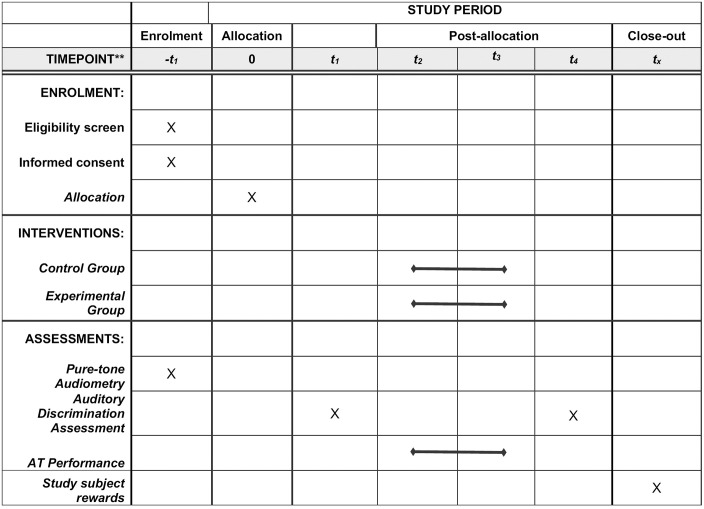
SPIRIT schedule or enrollment, interventions and assessments for Clinical Trials.

A total of 40 participants will be recruited and enrolled in the study, as described in Fig 1, predicting a sample rejection of ~10%. Each of them will be randomly assigned to one of two groups (control group or experimental group) depending on the training methodology they will receive. Sample size for each group (non-game-based ADT and game-based ADT) is 20 individuals each. Assignments of each participant will be randomized using simple randomization 1:1 allocation ratio to select the group for the training methodology, avoiding bias on trials.

No blinding will be involved within participants nor personnel who analyze data, since they must know which group they belong to, so instructions can be explained and clearly understood.

Participants will be recruited from the Tecnologico de Monterrey student population by local advertising. They will be invited to fill-in a survey to determine and filter, considering eligibility criteria, the participants that are able to be part of the research sample. These selected people will participate in the ADT sessions for a total of 15 hours, informing them about the activities to be done and the main objective. They will be given the informed consent to be read and signed before audiologic tests. After consent and tests are fulfilled, they will be receiving a demo of the training methodology they were assigned to and a tutorial.

Inclusion criteria are the following: Both genre, age between 18 and 30 years old. Volunteers with normal hearing pure-tone thresholds 25 dB HL across frequencies 125–8000 Hz previously evaluated by air-conduction and bone-conduction audiometry.

Exclusion criteria are the following: Participants that do not sign the informed consent, that present hearing loss or impairment pure-tone 25 dB HL, that cannot fulfill the required time for the AT, that is pregnant or that is frequently consuming medicines that can alter the participant state, that is currently enrolled in another auditory training or ear training intervention.

### Materials

**Fig 2:**
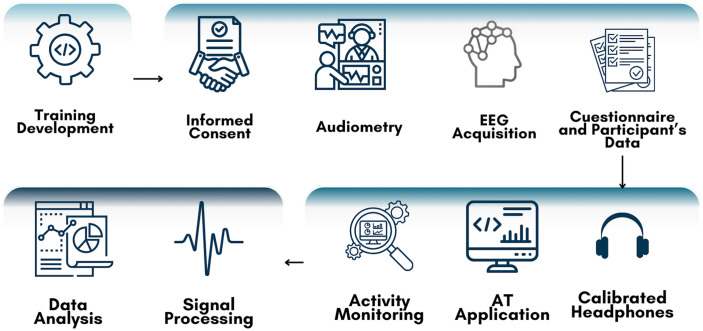
AT materials. Study materials are shown as to be used in each stage of research. First stage is the development of the training tools in Unity. Second stage is the application of auditory assessment for auditory processing screening which will be managed in two sessions of 50 minutes. Third stage refers to the application of auditory training during 15 hours. Fourth stage refers to the data management.

Materials to be used in each of the four main stages, as shown in Fig 2, are described as follows.

ADT Game will be developed to enhance user experience and favor attention through task engagement. ADT Game will consist in basic sound features discrimination training (pitch, intensity, duration and localization). Each participant will use personal computer and calibrated headphones to receive training. For auditory game-based and non-game-based training, Unity Software and IDE Visual Studio Code (C#) will be used to generate stand-alone applications for both Windows and Mac OS.

For neuro-audiological assessments audiometer Interacoustics AD226 with audiometric headset DD45 and B71 Bone conductor will be used to conduct pure-tone tests and Unicorn Hybrid Black 8-channel EEG (250 Hz), with electrode placement using the 10–20 system, and calibrated headphones Senheisser SRH440 (10–22,000 Hz) will be used to manage AEPs.

A brief basic auditory discrimination performance questionnaire (ADPQ) was designed for addressing participant’s discrimination and complementary conditions during and after each of experimental acoustic features under attentive processes. Questionnaire (I-CVI > 0.875) consists of 7 items evaluating in a 4-point likert scale the performance status of the participant (perceived state and perceived performance). This questionnaire helps the present study to have a perceptual participant’s discrimination response and to reduce participant’s state bias during the assessment.

For acquisition software, OpenVibe will be used to record EEG signals and Built-in App developed in Super Collider will be used for sound reproduction using a self-designed application to make easier the adjustment of multiple acoustic feature paradigms.

For data software, EEGLAB Matlab toolbox will be used for processing and analysis of EEG recorded data. Matlab software will be used for data analysis obtained from participants performance during training and as result of the auditory assessments.

### Study design & setting

This work is divided in three instances: 1) development and validation of the digital game, 2) implementation and evaluation of the ADT game performance in auditory improvement, 3) comparison between both methodologies.

The first stage consists of serious game development. This stage is divided into two sub-stages. First one is back-end development (auditory tasks) and tests and second one is front-end development (interactive elements) and tests, additionally both are having their respective correction sub-stages.

**Fig 3:**
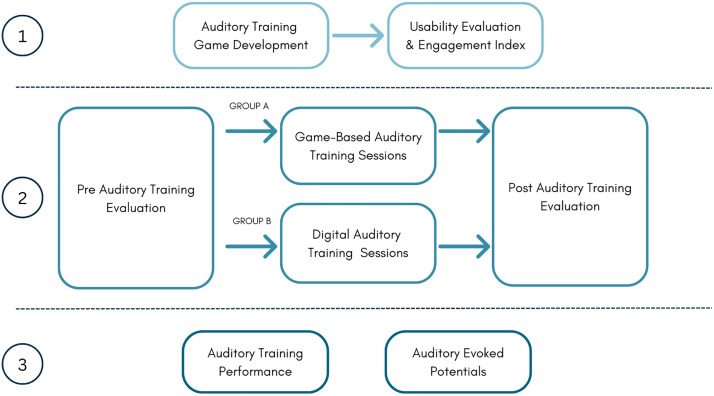
Protocol steps. 1) Game-Based AT is first developed and validated through perceived usability evaluation questionnaires. 2) Assessments are done before and after AT is implemented for two sample groups. 3) Finally AT effectiveness is measured and compared through groups performance and AEPs.

Second stage consists in the implementation of the game-based ADT, as described in Fig 3. In 30-minutes sessions (15 hours total), the 40 participants will be treated by ADT; 20 participants will use the game-based and 20 participants will be treated by a non-game-based training procedure with the same four modules in both cases. Both procedures will locally save training data in participant computer, which will be gathered each week.

The participant will be introduced to the study, explaining to them the informed consent and the confidentiality criteria. Participants will be given their audiometry results and a beginner game kit, also they will receive an online-shop gift card ($5-$25 us) if they finish the training levels satisfactorily, based on constant and consistent game playing, meaning they accomplish daily training and completely succeed in all training levels. Participants will also receive hearing health information after intervention and the option of scheduling a medical appointment with the otorhinolaryngologist and/or audiologist if they desire.

Auditory assessments will be conducted in noise-controlled rooms within university buildings or hospitals. Eligibility screen, informed consent and pure tone audiometry will be carried before allocation. Procedure to follow will be explained to them, beginning the pure-tone audiometry (air and bone) procedure. The participant will be indicated to raise the hand when they start to listen to the stimuli.

Then, they will be introduced to the electrode placements. Electrodes (Fz, F3, F4, Cz, C3, C4, T7 & T8) will be placed, and conductive gel will be applied to reduce the electrode-skin impedance. Calibrated headphones will be placed carefully. The participant will be asked to remain seated and still during evaluation. This process will be applied for the pre and post training assessment.

**Fig 4:**
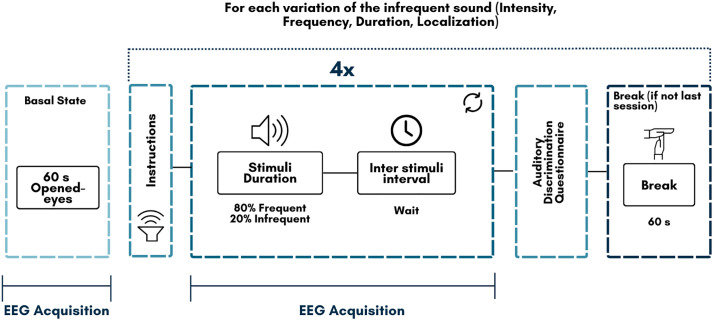
Auditory discrimination paradigm procedure. Basal state is only recorded at the beginning of the session. In each of the feature variation the rest of the procedure is repeated.

As shown in Fig 4. basal state will be recorded for 60 s in opened and closed eyes conditions. Then oddball paradigm will be used to assess auditory discrimination in pitch, intensity, duration and localization of the stimuli. P300 & Mismatch Negativity Potentials will be assessed by this paradigm in attentional and non-attentional conditions.

Sound system used for stimuli is integrated by a sound interface (DAC/ADC) with supra-aural Shure SRH 440’ headphones system calibrated to audiometric reference of 1kHz to 70 dB HL. Sound stimuli consists in pure tones lasted with 100 ms duration and with a 900 ms ISI as the standard stimuli. Deviant stimuli vary in each of the four testing sound features (pitch, intensity, duration and localization). EEG event related activity will be processed in Matlab with EEGLab, for preprocessing and epoching (−200ms, 600ms)”.

For the AT, digital tools will be shown to participants, they will be instructed in how to use it, and main features will be explained. They will be told to use it daily for 30 minutes indicating the modules to evaluate in each week sessions. Game data (training time, response time and correct responses) will be gathered for participants after each week to register in-game indicators. They will be asked not to do more or less time than indicated, if indication is not followed, they will not be eligible to continue participating. An extra explanatory video will be provided with a user manual.

### AT experimental design

AT for both groups will contain the same four modules related to auditory discrimination tasks. Each module consists of one basic acoustic feature (pitch, intensity, duration, and localization). Participants will solve pure tone discrimination tasks identifying feature differences between acoustic stimuli A from B (reference tone), and answer whether stimuli feature was higher or lower. Reference tones are centered around 0.5, 1, 2 and 4 Khz with duration of 100 ms (fade in and fade out of 5 ms). Both groups will have auditory training for an equivalent of 15 hours, with daily monitoring data and gathered every week. Training will be applied with measured and calibrated headphones given to the participants.

#### Group A (Control).

AT for non-game-based group will consist in a simple graphic user interface. Instructions will be displayed, and once the features are selected, the user will start training getting basic feedback of wrong and right answers.

#### Group B (Experimental).

AT for game-based group will have game elements and competing resources, such as game objective, game characters and visuals, CPU competition players, feedback, time and lives management.

AT methodologies to be used are non-game based auditory training and game-based auditory training. Each tool is applied as indicated in the SPIRIT checklist in Fig 1.

### Ethical considerations & data management

This study conforms to the declaration of Helsinki and has been approved by the Ethical Committee Instituto Tecnologico y de Estudios Superiores de Monterrey Engineering School with ID EHE-2023-11. Research was also registered with the International Standard Randomized Controlled Trial Number ISRCTN43661994 on January 26^th^ 2024, for research transparency. Recruitment of participants in the study is completely free and voluntary. Enrollment will start on January 2025, expecting the end of data collection by the end of July 2025. Before any assessment participants will be explained about research procedures and written informed consent will be delivered and signed by each participant, who can ask for a copy if it is desired.

For correct management of the participants’ information the following points will be accomplished all during the assessment sessions period.

- Recruited participants will be completely voluntary.
- Assessment registers will be done in Tec de Monterrey so it will not be necessary to attend to another place.
- Participation will be completely free. Material will be proportionated to applicants.
- Individual evaluations and assessments will be given to volunteers.
- Volunteers will receive the information and explanation of each step before, during and after the training to guarantee uniformity and informed participation.
- Participants will sign an informed consent format before the session begins. Participants will receive a copy of the consent. This information will be kept by the applicants and can be consulted for any investigation purpose.
- Participants’ identity will remain anonymous. Participants’ information will be treated with complete confidentiality.
- Applicants will count with disposition towards the participants for evaluation purposes.
- Participants’ information will be delivered only to the institution and legal authorities if required.

All registers and assessments including audiometry and AEPs are non-invasive, which will not place participants at higher risk other than listening. To minimize risk of placing participants to high sound pressure level for a long period of time, sound pressure level will be fixed within the equipment to use so that they would not exceed suggested sound levels.

Any medical or clinical occurrence related or not with the research procedure, will be managed as an adverse event, and treated or reported according to institutional health services protocol, mainly considering participant safety and health. Adverse events will be further evaluated for participant continuity in the research experiment.

### Expected outcomes & analysis

The primary outcome measures are related to neuro audiological changes in auditory processing. AEPs to be obtained are Mismatch Negativity and P3 components (continuous). Additionally, number of discrimination trials (discrete) will be registered. The secondary outcome measures are the in-game indicators such as time in each activity (continuous), accuracy (discrete) and attempts (discrete) to relate directly changes in audiological measures and game performance. The expected outcome of ADPQ is to increase the average score, that indicates a better perceived state and performance in auditory discrimination tasks that would be correlated with the in-game indicators and the electrophysiological measurements. The expected comparison outcome for AEP, it is expected an increase in evoked potentials amplitude and a decrease in latencies. For the in-game indicators it is expected to increase accuracy answers and decrease tasks’ solving time.

Once obtained and processed KPIs and discrimination threshold results, data will be analyzed with the following tools. Each of them will compare before and after auditory training KPIs and AEPs tests.

A 2-way analysis of variance (ANOVA) with repeated measures will be done considering the following factors.

- For pre & post training measurements to determine individual performance of each methodology and contrast the improvement of each (within factors).
- For training group. Both methodologies have a similar distribution of activities to accomplish withing the training. Each group will be evaluated to determine if some activity yielded to a more significant difference in the overall performance (between factors).
- Finally, and mainly, for AT methodology groups to analyze and compare the final performance between the game-based experimental groups and the non-game-based control group and conclude if there is a significant difference between them.

The following cases are proposed to test main 2-way ANOVA with repeated measures assumptions (normality, sphericity and equal variance) [37,38]. Normal distribution will be addressed using a histogram and Shapiro-Wilk test. If no normal distribution is found, data transformation (log or sqrt) will be conducted to repeat the normality test. Treating Type I errors of heteroscedasticity, sphericity would be addressed through a Maulchy’s test. If no sphericity was found, Greenhouse-Geisser correction would be conducted to adjust degrees of freedom and repeat sphericity test. Since this study within-subjects factor (repeated measures) is only having two measurements, sphericity assumption is not required due to no covariance comparisons. If normal distribution is not corrected, repeated measures ANOVA non-parametric equivalent test will be used (Friedman’s test). An additional Barlett’s test would be done for equal variance assumption if ANOVA normality is confirmed. If equal variance is violated, robust ANOVA may be considered.

Bonferroni post-hoc test will be conducted, using multiple t-tests times p-values (to avoid increase in alpha error). Analysis software to be used is Minitab with a level of significance of 0.05.

Possible spillover effects that could bias primary outcome measures are related to 3 main conditions:

- Participants discrimination performance: This effect is considered through a discrimination performance questionnaire that the participant answer to address biases during tests of outcome measures.
- Participants health condition (auditory pathways related): This effect is addressed through rescheduling the tests when hearing health condition is better.
- Participants simultaneous training: This effect is addressed in the inclusion criteria where no additional formal auditory training is performed by the participant at the time of intervention.

## Discussion

International guidelines suggest auditory training as an important part of the aural rehabilitation process supplementing hearing loss management best practices [8,39]. Several reports in the last decades have shown that AT can benefit both hearing impairment and normal hearing population to improve sensory and perceptual auditory responses. As mentioned in State of Art, there has also been some research in the last decade about computer-based and game-based AT. Some of these have developed their own software and some others have used commercial programs. Most research has been focused on demonstrating improvement in cognitive-auditory processing assessments and tasks, mainly oriented in speech recognition and dichotic-based training.

Very little was found in the literature about validation criteria and usability evaluation for digital tools such as games, apps, or web software that in fact contribute to the overall benefit of the AT and that answer the question of how is game experience and training engagement related to improvement in AT performance? Some interesting findings have been presented by Whitton [21] that tried to develop a new proposal of AT tasks, although no validation of the methodology in terms of the framework was done.

Another interesting finding was done by Larrea-Mancera et al. [15] in the gamified AT approach integrating perceptual learning and video-game play for speech competition with a mixed training with three activity modules. Designing the training based on game experience presented in an obstacle avoiding or “runner” format game, based on motivation for broad improvements in visual and cognitive processing skills enhancement by gamification.

Lastly and following this line for auditory computer-based training validation, Tuz [16] explored the importance of the user program evaluation through usability questionnaires and established a consistent antecedent for subjective validation of the methodology. Although results of the AT performance were not published yet, it suggested a starting point for game-based auditory training user evaluation.

All these studies still did not explore the performance of training directly on discrimination thresholds estimation through audio neurological procedures to ensure a change not only on brain plasticity but in auditory processing, frequency response and neural timing.

This study tries to find a relationship experience-performance in AT, specifically, addressing game elements and engagement in auditory processing improvement.

As part of some issues considered during the performing of the study, limitations found are the following:

- Restriction by installations and equipment to be used calibration and justification of study setting and materials will be included as part of the final publications.
- Study design depends on participants constant and correct usage of auditory training methodology. Since daily monitoring of 50 participants is not possible, monitoring will be done every week.
- Sample size is above as the calculated sample size considering participants withdrawal of 10%, although withdrawal rate could be different.

With scientific dissemination purposes, once analysis is concluded research it is intended to publish results as original research paper, analyzing auditory processing discrimination results through AT performance and electrophysiological measurements. Research would be published in journals related to auditory health, auditory neuroscience, or physiological acoustics approximately by the end of 2025. With transparency purposes participants’ measurements data will also be intended to be published by the same date in database repository, so it can be available for further research.

Once evaluated the generality of the study, amendments considered for future research are the following:

- To have more measurements in time and monitor participants training continuity and to have a follow up with one last measurement after some specific time after training.
- To meet one more inclusion criterion based on auditory processing tests to determine if there exists any irregularity among participants.
- To have specialized medical equipment to obtain other participants audiological assessments.
- To apply longer AT sessions and during more time to measure improvement to long-time exposure effect.
- To develop a higher number of game-based audiologic tasks and levels to have a broader evaluation.
- To change recruitment target to hearing-loss population, so once tool and methodology are validated new improvements can be measured in this population.

With this research it is expected that auditory processing improvement can be addressed by the efficient use of engaging computerized resources such as game-based training tools, permitting further works that may benefit and contribute to hearing impairment and auditory disorder populations potentially in auditory discrimination, sound localization and lateralization, auditory pattern recognition, and temporal processing.

## References

[1] Deafness and hearing loss. [cited 2025 Feb 17]. Available from: https://www.who.int/news-room/fact-sheets/detail/deafness-and-hearing-loss

[2] Paulraj MP, Subramaniam K, Yaccob SB, Adom AHB, Hema CR. Auditory evoked potential response and hearing loss: a review. Open Biomed Eng J. 2015;9:17–24. doi: 10.2174/1874120701509010017 25893012 PMC4391208

[3] Oshima K, Suchert S, Blevins NH, Heller S. Curing hearing loss: patient expectations, health care practitioners, and basic science. J Commun Disord. 2010;43(4):311–8. doi: 10.1016/j.jcomdis.2010.04.002 20434163 PMC2885475

[4] Henshaw H, Ferguson MA. Efficacy of individual computer-based auditory training for people with hearing loss: a systematic review of the evidence. PLoS One. 2013;8(5):e62836. doi: 10.1371/journal.pone.0062836 23675431 PMC3651281

[5] Con discapacidad auditiva, 2.3 millones de personas: Instituto Nacional de Rehabilitación | Secretaría de Salud | Gobierno | gob.mx. [cited 2025 Feb 17]. Available from: https://www.gob.mx/salud/prensa/530-con-discapacidad-auditiva-2-3-millones-de-personas-instituto-nacional-de-rehabilitacion?idiom=es

[6] En México, tres de cada mil nacidos presentarán discapacidad por sordera | Secretaría de Salud | Gobierno | gob.mx. [cited 2025 Feb 17]. Available from: https://www.gob.mx/salud/prensa/046-en-mexico-tres-de-cada-mil-nacidos-presentaran-discapacidad-por-sordera

[7] Hirt Z, Kohanzadeh A, Gibber M. Sensorineural hearing loss as a complication of COVID-19 and the COVID-19 vaccine. Cureus. 2023;15(10):e47582. doi: 10.7759/cureus.47582 38021934 PMC10665765

[8] Turton L, Souza P, Thibodeau L, Hickson L, Gifford R, Bird J, et al. Guidelines for best practice in the audiological management of adults with severe and profound hearing loss. Semin Hear. 2020;41(3):141–246. doi: 10.1055/s-0040-1714744 33364673 PMC7744249

[9] Bower C, Reilly BK, Richerson J, Hecht JL, Committee on Practice & Ambulatory Medicine, Section on Otolaryngology–Head and Neck Surgery. Hearing assessment in infants, children, and adolescents: recommendations beyond neonatal screening. Pediatrics. 2023;152(3):e2023063288. doi: 10.1542/peds.2023-063288 37635686

[10] Kwalzoom Longpoe P. Effect of auditory training intervention on auditory perception problem of children with perceptual disorders in Nigeria. JRTDD. 2020;:42–53. doi: 10.26407/2020jrtdd.1.27

[11] Nisha KV, Uppunda AK, Kumar RT. Spatial rehabilitation using virtual auditory space training paradigm in individuals with sensorineural hearing impairment. Front Neurosci. 2023;16:1080398. doi: 10.3389/fnins.2022.1080398 36733923 PMC9887142

[12] Barton B, Brewer AA. Attention and working memory in human auditory cortex. In: The human auditory system - basic features and updates on audiological diagnosis and therapy. IntechOpen; 2020. doi: 10.5772/intechopen.85537

[13] Turan Z, Meral E. Game-based versus to non-game-based: the impact of student response systems on students’achievements, engagements and test anxieties. Inform Educ. 2018;17(1):105–16. doi: 10.15388/infedu.2018.07

[14] Brice S, Almond H. Is teleaudiology achieving person-centered care: a review. Int J Environ Res Public Health. 2022;19(12):7436. doi: 10.3390/ijerph19127436 35742684 PMC9224155

[15] COVID-19 cases | WHO COVID-19 dashboard. [cited 2025 Feb 17]. Available from: https://data.who.int/dashboards/covid19/cases?n=c

[16] Meng X, Wang J, Sun J, Zhu K. COVID-19 and sudden sensorineural hearing loss: a systematic review. Front Neurol. 2022;13:883749. doi: 10.3389/fneur.2022.883749 35572936 PMC9096262

[17] Skoe E. Hearing: the future of sensory rehabilitation? Curr Biol. 2017;27(21):R1163–5. doi: 10.1016/j.cub.2017.09.053 29112869

[18] Ferguson MA, Henshaw H. Auditory training can improve working memory, attention, and communication in adverse conditions for adults with hearing loss. Front Psychol. 2015;6:556. doi: 10.3389/fpsyg.2015.00556 26074826 PMC4447061

[19] de Larrea-Mancera ESL, Philipp MA, Stavropoulos T, Carrillo AA, Cheung S, Koerner TK, et al. Training with an auditory perceptual learning game transfers to speech in competition. J Cogn Enhanc. 2022;6(1):47–66. doi: 10.1007/s41465-021-00224-5 34568741 PMC8453468

[20] Tuz D, Isikhan SY, Yücel E. Developing the computer-based auditory training program for adults with hearing impairment. Med Biol Eng Comput. 2021;59(1):175–86. doi: 10.1007/s11517-020-02298-3 33400137

[21] Shepard KN, Kilgard MP, Liu RC. Experience-dependent plasticity and auditory cortex; 2013. p. 293–327. doi: 10.1007/978-1-4614-2350-8_10

[22] Coelli S, Sclocco R, Barbieri R, Reni G, Zucca C, Bianchi AM. EEG-based index for engagement level monitoring during sustained attention. Proceedings of the Annual International Conference of the IEEE Engineering in Medicine and Biology Society, EMBS. Institute of Electrical and Electronics Engineers Inc; 2015. p. 1512–5. doi: 10.1109/EMBC.2015.7318658 26736558

[23] Smiderle R, Rigo SJ, Marques LB, Peçanha de Miranda Coelho JA, Jaques PA. The impact of gamification on students’ learning, engagement and behavior based on their personality traits. Smart Learn Environ. 2020;7(1). doi: 10.1186/s40561-019-0098-x

[24] Preminger JE. Auditory training: consideration of peripheral, central-auditory, and cognitive processes. Semin Hear. 2015;36(4):197–8. doi: 10.1055/s-0035-1564459 27587908 PMC4954541

[25] Stewart HJ, Martinez JL, Perdew A, Green CS, Moore DR. Auditory cognition and perception of action video game players. Sci Rep. 2020;10(1):14410. doi: 10.1038/s41598-020-71235-z 32873819 PMC7462999

[26] Whitton JP, Hancock KE, Shannon JM, Polley DB. Audiomotor perceptual training enhances speech intelligibility in background noise. Curr Biol. 2017;27(21):3237-3247.e6. doi: 10.1016/j.cub.2017.09.014 29056453 PMC5997394

[27] Sailer M, Hense JU, Mayr SK, Mandl H. How gamification motivates: an experimental study of the effects of specific game design elements on psychological need satisfaction. Comput Hum Behav. 2017;69:371–80. doi: 10.1016/j.chb.2016.12.033

[28] Picinini T de A, Sperança S, Pereira LD. Acoustically controlled binaural auditory training with vocal duets: assessment and effectiveness. Clinics (Sao Paulo). 2021;76:e2085. doi: 10.6061/clinics/2021/e2085 33787671 PMC7955147

[29] Joshi YB, Gonzalez CE, Molina JL, MacDonald LR, Min Din J, Minhas J, et al. Mismatch negativity predicts initial auditory-based targeted cognitive training performance in a heterogeneous population across psychiatric disorders. Psychiatry Res. 2023;327:115215. doi: 10.1016/j.psychres.2023.115215 37406367 PMC13250892

[30] Henshaw H, Ferguson MA. Efficacy of individual computer-based auditory training for people with hearing loss: a systematic review of the evidence. PLoS One. 2013;8(5):e62836. doi: 10.1371/journal.pone.0062836 23675431 PMC3651281

[31] Stropahl M, Besser J, Launer S. Auditory training supports auditory rehabilitation: a state-of-the-art review. Ear Hear. 2020;41(4):697–704. doi: 10.1097/AUD.0000000000000806 31613823

[32] Nisha KV, Kumar AU. Effects of spatial training paradigms on auditory spatial refinement in normal-hearing listeners: a comparative study. J Audiol Otol. 2022;26(3):113–21. doi: 10.7874/jao.2021.00451 35196448 PMC9271736

[33] Dornhoffer JR, Reddy P, Ma C, Schvartz-Leyzac KC, Dubno JR, McRackan TR. Use of auditory training and its influence on early cochlear implant outcomes in adults. Otol Neurotol. 2022;43(2):e165–73. doi: 10.1097/MAO.0000000000003417 34772887 PMC8752503

[34] Chae SR, Bahng J. Effective duration of auditory training for speech perception in hearing-impaired listeners: a systematic review and meta-analysis. Clin Arch Commun Disord. 2023;8(3):192–210. doi: 10.21849/cacd.2023.01004

[35] Lelo de Larrea-Mancera ES, Stavropoulos T, Carrillo AA, Cheung S, He YJ, Eddins DA, et al. Remote auditory assessment using Portable Automated Rapid Testing (PART) and participant-owned devices. J Acoust Soc Am. 2022;152(2):807. doi: 10.1121/10.0013221 36050190 PMC9355663

[36] Olson AD. Auditory training at home for adult hearing aid users. University of Kentucky; 2010.

[37] Smith PF. A guerilla guide to common problems in “neurostatistics”: essential statistical topics in neuroscience. J Undergrad Neurosci Ed. 2017. Available from: https://www.researchgate.net/publication/322744618 PMC5777851 29371855

[38] Muhammad LN. Guidelines for repeated measures statistical analysis approaches with basic science research considerations. J Clin Invest. 2023;133(11):e171058. doi: 10.1172/JCI171058 37259921 PMC10231988

[39] Aural Rehabilitation Clinical Practice Guideline Development Panel, Basura G, Cienkowski K, Hamlin L, Ray C, Rutherford C, et al. American Speech-Language-Hearing Association clinical practice guideline on aural rehabilitation for adults with hearing loss. Am J Audiol. 2023;32(1):1–51. doi: 10.1044/2022_AJA-21-00252 36374028

